# Anatomical-Based Filler Injection Diagnosis to Treatment Techniques: Infraorbital Groove and Hollowness

**DOI:** 10.3390/life15020237

**Published:** 2025-02-05

**Authors:** Gi-Woong Hong, Wonseok Choi, Song-Eun Yoon, Jovian Wan, Kyu-Ho Yi

**Affiliations:** 1 Samskin Plastic Surgery Clinic, Seoul, Republic of Korea; 2 V Plastic Surgery, Daegu, Republic of Korea; 3 Brandnew Aesthetic Surgery Clinic, Seoul, Republic of Korea; 4 Medical Research, Wonju, Republic of Korea; 5 Division in Anatomy and Developmental Biology, Department of Oral Biology, Human Identification Research Institute, BK21 FOUR Project, College of Dentistry, Yonsei University, Seoul 03722, Republic of Korea; 6 You & I Clinic (Mokdong), Seoul, Republic of Korea

**Keywords:** Infraorbital region, tear trough, hyaluronic acid, facial aging, dermal fillers

## Abstract

Infraorbital groove and hollowness are common aging-related anatomical changes in the periorbital region, leading to a sunken and tired appearance. These conditions are caused by the progressive loss of volume in the deep fat pads, including the sub-orbicularis oculi fat (SOOF) and malar fat, along with skin thinning and decreased elasticity. Filler injections, particularly hyaluronic acid-based fillers, are the preferred treatment to restore volume and smooth the under-eye area. Proper diagnosis, understanding of anatomical variations, and accurate injection techniques are essential to avoid complications and achieve natural, esthetically pleasing outcomes. Treatments should consider the patient’s unique anatomy and potential adjunctive procedures to ensure balanced and harmonious facial rejuvenation.

## 1. Introduction

Infraorbital groove and hollowness are common anatomical changes that occur with aging, leading to a sunken and fatigued appearance in the under-eye area. These conditions are characterized by volume loss in the fat pads and structural shifts in the skin and soft tissue layers, particularly in the infraorbital and malar regions. The infraorbital groove, also referred to as the tear trough, forms as a pronounced indentation along the lower boundary of the orbital bone, while infraorbital hollowness manifests as a more widespread depression beneath the eyes. Together, these changes contribute to the appearance of deep grooves, dark circles, and sagging skin, creating a tired and aged look. Proper diagnosis and treatment, particularly using filler injections, can restore volume and enhance the esthetic appearance of this area [1,2,3,4,5,6,7,8,9,10].

The infraorbital groove and hollowness are distinct yet interrelated phenomena influenced by individual anatomy and aging processes. In the youthful face, a balance of fat pads provides support and contour, but with time, these fat compartments shift and atrophy, leading to structural changes. Tear trough deformities, commonly found in the infraorbital region, may coexist with nasojugal grooves and other midface depressions, further complicating the under-eye appearance. Filler injections are a key technique for addressing these concerns, offering a non-invasive approach to restoring volume, improving skin support, and achieving a rejuvenated look. A thorough understanding of the anatomical variations and accurate injection techniques are essential for achieving effective and natural results.

## 2. Material and Methods

A comprehensive literature search was conducted using keywords such as “Infraorbital region”, “tear trough”, “hyaluronic acid”, “facial aging”, and “dermal fillers” across MEDLINE, PubMed, and Ovid databases. The search focused on identifying studies published on the anatomy, diagnosis, and treatment techniques specific to infraorbital groove and hollowness, including the use of hyaluronic acid fillers. Studies were selected based on criteria like detailed descriptions of filler injection methods, anatomical dissections, and clinical outcomes in treating periorbital hollowness. Studies were further reviewed for criteria including double-blinding, sample size adequacy, randomization, use of control groups, and objective outcome measures. Following these filters, studies were organized in accordance with the Oxford Centre for Evidence-Based Medicine evidence hierarchy. After applying the inclusion criteria to the initial search results, a total of 125 studies were identified. Following a thorough review, 110 studies were excluded due to insufficient methodological detail, small sample sizes, lack of control groups, or absence of objective outcome measurements. Consequently, 15 studies met the criteria and were included in the review, forming the basis for discussing anatomical-based filler injection techniques for infraorbital groove and hollowness.

## 3. Infraorbital Groove and Hollowness

**Figure 1:**
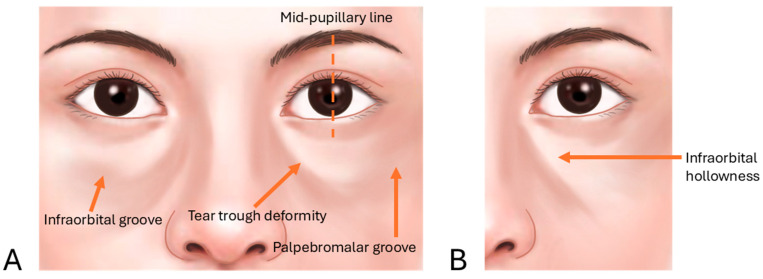
Infraorbital groove (tear trough deformity and palpebromalar groove, (**A**)) and hollowness (**B**).

Infraorbital groove, often referred to as the tear trough, is a pronounced indentation along the lower orbital rim, creating a shadowed area beneath the eyes and contributing to a fatigued or aged appearance. When this feature extends into a broad, sunken area, it is termed infraorbital hollowness. These conditions develop due to age-related changes in the skin, fat pads, and supporting structures around the eyes, where the malar fat pad (responsible for youthful fullness in the cheeks) diminishes and descends, while the infraorbital area appears hollow (Figure 1) [8,11,12].

**Figure 2:**
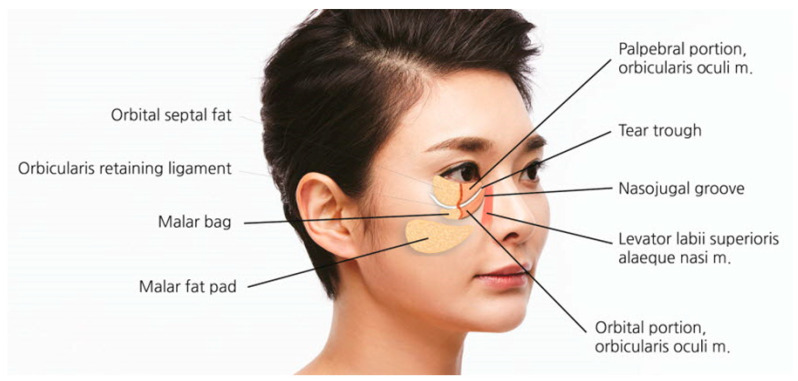
Tear trough deformity and nasojugal groove.

Anatomically, the infraorbital groove forms where skin thickness, fat distribution, and ligament attachments, such as the tear trough ligament (TTL) and orbicularis retaining ligament (ORL), create a tethered effect. This junction is situated between the palpebral and the orbital portions of the orbicularis oculi muscle, aligning with the cephalic border of the malar fat pad approximately 2–3 mm below the orbital rim. These features contribute to infraorbital shadowing and emphasize the need for careful filler placement (Figure 2) [8,12,13].

**Figure 3:**
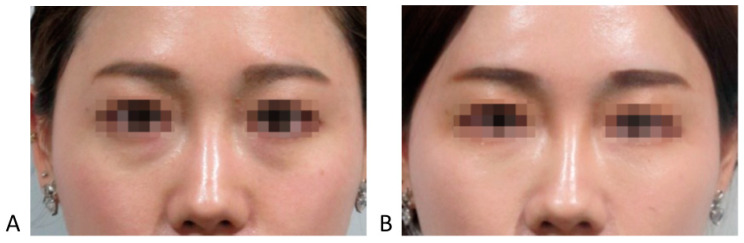
Before and after treatment of the tear trough deformity.

**Figure 4:**
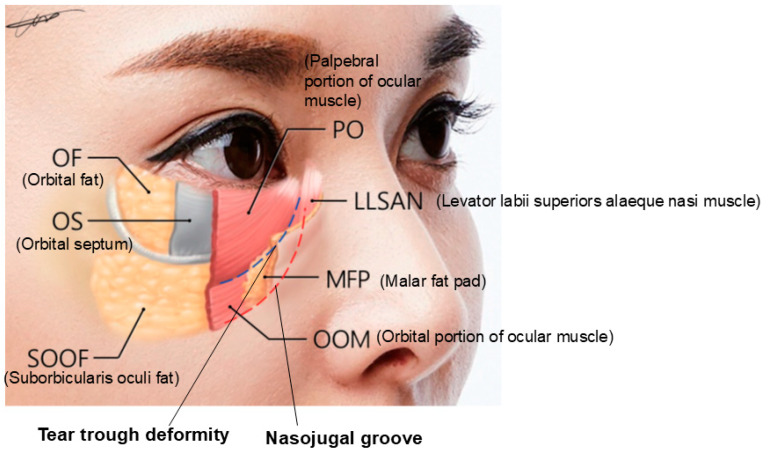
Location of the tear trough deformity and nasojugal groove [15].

**Figure 5:**
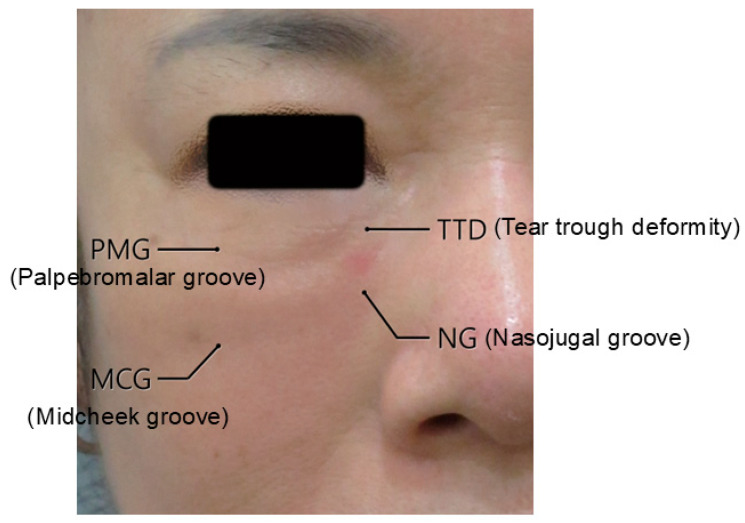
The grooves of the infraorbital region.

Infraorbital grooves can manifest as a tear trough (medial infraorbital groove) or extend laterally as a palpebromalar groove. Koreans tend to display a prominent infraorbital groove without extensive hollowness due to thicker skin and soft tissues, unlike many Caucasians [8,11]. The tear trough deformity primarily affects the medial one-third of the lower eyelid and may extend laterally with age, while the nasojugal groove extends from the nose toward the cheek, forming a less pronounced line beneath the orbital rim [8,12,14]. Effective filler placement requires precise identification of these landmarks to avoid complications, especially where major vessels like the infraorbital artery and facial vein traverse the area (Figure 3, Figure 4 and Figure 5) [11,14].

**Figure 6:**
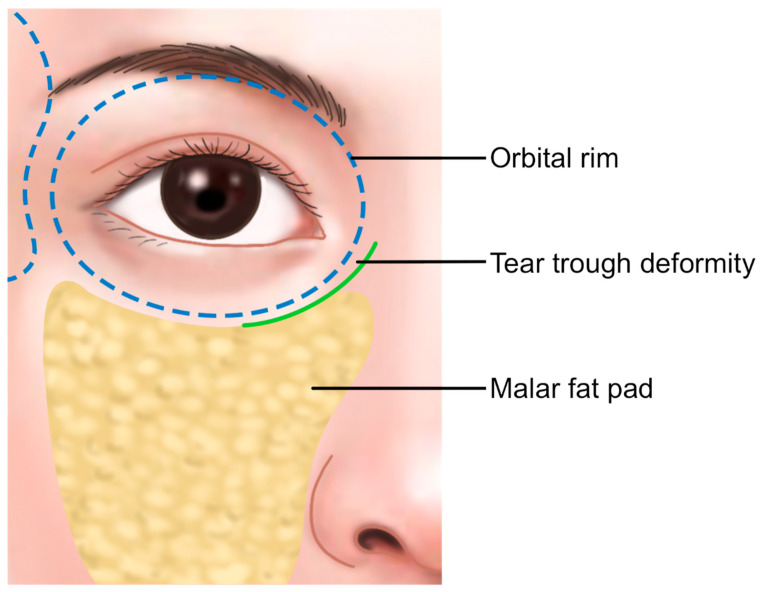
Position of the malar bag (malar fat pad).

**Figure 7:**
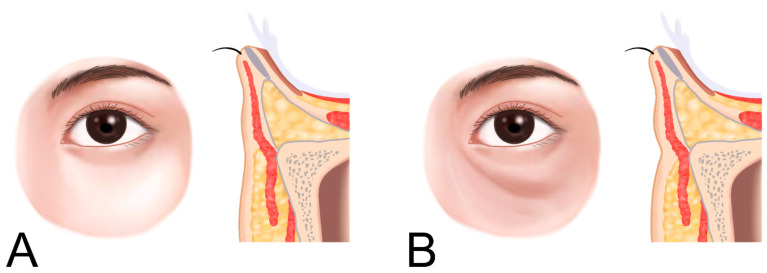
Appearance of infraorbital hollowness. Panel (**A**) shows normal conditions, while panel (**B**) illustrates hollowness with soft tissue atrophy.

In cases with herniated infraorbital fat, carefully placed filler can reduce the appearance of infraorbital bulging without surgical intervention (Figure 6 and Figure 7).

Infraorbital hollowness can typically be classified into three categories based on its presentation [16]:

Class 1: Characterized by depression primarily in the medial orbit, often associated with the tear trough. Mild flattening of the central cheek may also be observed.

Class 2: The hollowing of the medial orbit becomes more pronounced, and the tear trough and the nasojugal groove, which is the medial portion of the midcheek groove, become more evident. In this stage, the infraorbital rim lateral to the limbus also starts to exhibit hollowness, often accompanied by a mild volume deficiency in the medial cheek and a mild flattening of the central cheek triangle.

**Figure 8:**
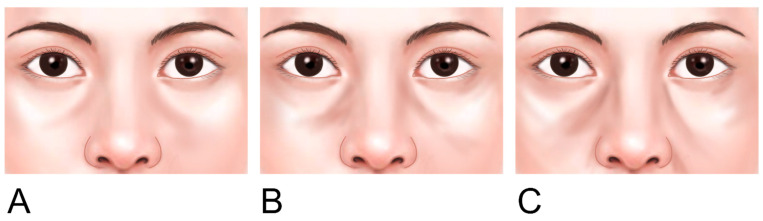
Classification of the infraorbital groove and hollowness. Panel (**A**) depicts Class I with hollowness limited to the medial orbit. Panel (**B**) shows Class II with medial and lateral depression, while Panel (**C**) illustrates Class III with full circumferential depression at the orbital rim.

Class 3: Marked by circumferential hollowing along the entire infraorbital rim. The lateral portion of the tear trough, known as the palpebromalar groove or lid-cheek junction groove, becomes visible, often accompanied by significant medial cheek volume deficiency, a reversal of the central cheek triangle, a pronounced nasojugal groove that extends laterally, an oblique midcheek groove, and the formation of a malar bag (Figure 8).

**Figure 9:**
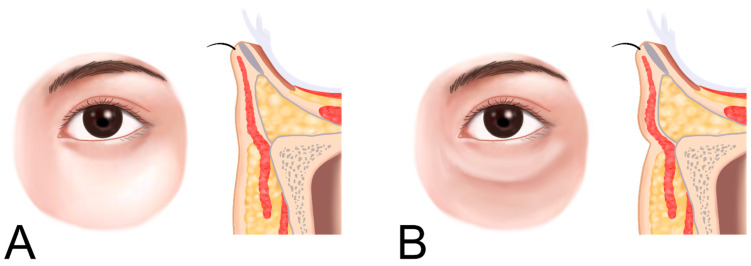
Groove or hollowness with herniation of the infraorbital fat. Normal condition (**A**) and herniation of infraorbital fat (**B**).

**Figure 10:**
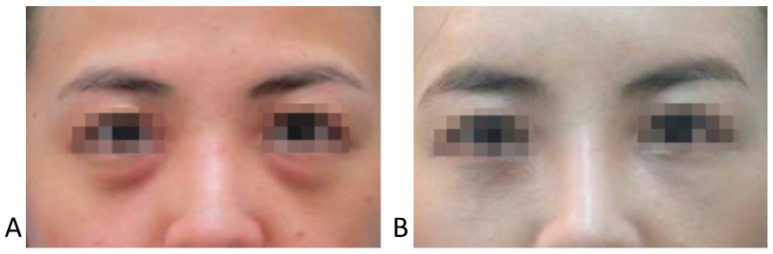
Before (**A**) and after (**B**) treatment of the tear trough deformity with herniation of infraorbital fat.

In Caucasians, the presence of a tear trough or infraorbital hollowness is often accompanied by thinning and hollowing of the skin in the palpebral portion of the lower eyelid. In contrast, East Asians frequently exhibit bulging of the orbital fat, which makes the palpebral portion of the lower eyelid appear more pronounced. In such cases, where the skin retains sufficient elasticity and is not excessively sagging, filler (L’orient, Joonghun Pharmaceutical, Seoul, Republic of Korea) injections can be used to fill the hollowed areas, creating the appearance of reduced orbital fat bulging, even without the need for surgical removal of the orbital fat (Figure 9 and Figure 10).

### 3.1. Anatomical Considerations

**Figure 11:**
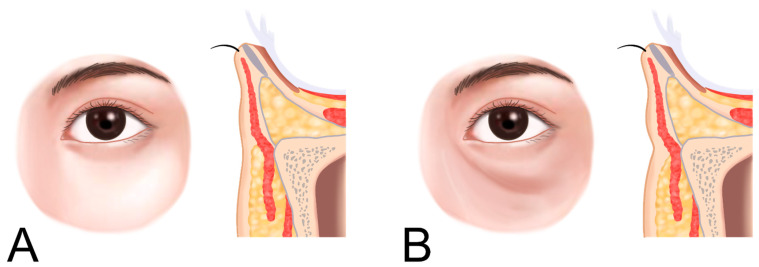
Location of tear trough deformity. Normal condition (**A**) and tear trough deformity through atrophy of skin and subcutaneous fat in the suborbital margin (**B**).

The tear trough, as previously described, is situated at the junction between the palpebral and orbital portions of the orbicularis oculi muscle. It aligns with the cephalic border of the malar fat pad and is typically located 2–3 mm below the orbital rim (Figure 11) [17].

Historically, it was believed that the orbicularis oculi muscle attached directly to the bone in the medial tear trough area, while in the lateral tear trough area, the muscle’s attachment was ligamentous due to the presence of the orbicularis retaining ligament (ORL). However, a 2012 study by Dr. Wong and colleagues [13] revealed that the medial tear trough area also possesses a distinct ligament, termed the tear trough ligament (TTL), located beneath the groove, which tethers the skin in a manner similar to a ligament. This finding has since gained widespread acceptance.

Current research indicates that the causes of the tear trough are multifactorial. Primarily, the medial tear trough ligament and the lateral orbicularis retaining ligament are key contributors to the formation of the groove. Additionally, variations in skin thickness and texture at the lid-cheek junction, along with differences in the thickness of the subcutaneous fat layer, further accentuate the boundary. The presence of bulging orbital fat can make the groove even more pronounced (Table 1) [13,18,19].

**Figure 12:**
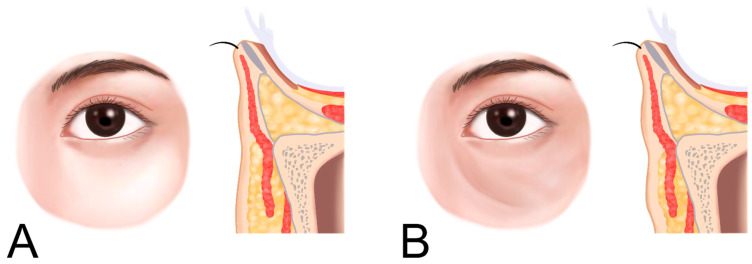
Location of nasojugal groove connected to midcheek groove. Normal condition (**A**) and nasojugal groove (**B**).

**Figure 13:**
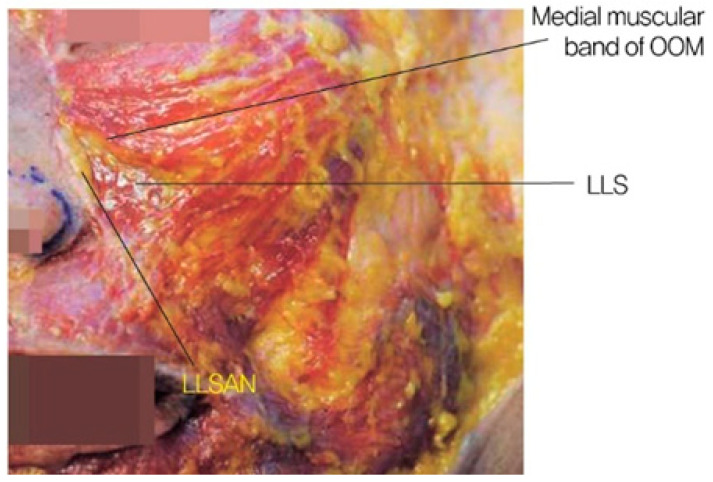
Medial muscular band of the orbicularis oculi muscle.

When the medial muscular band of the orbicularis oculi muscle is prominent, the contraction of the levator labii superioris alaeque nasi (LLSAN) and the levator labii superioris (LLS) muscles can accentuate the nasojugal groove, which constitutes the inner portion of the midcheek groove. This can result in a more pronounced and continuous line that extends to the outer midcheek groove (Figure 12) [20]. Cadaver studies have confirmed the presence of a pronounced medial muscular band within the orbicularis oculi and surrounding muscles, contributing to this effect (Figure 13) [21].

**Figure 14:**
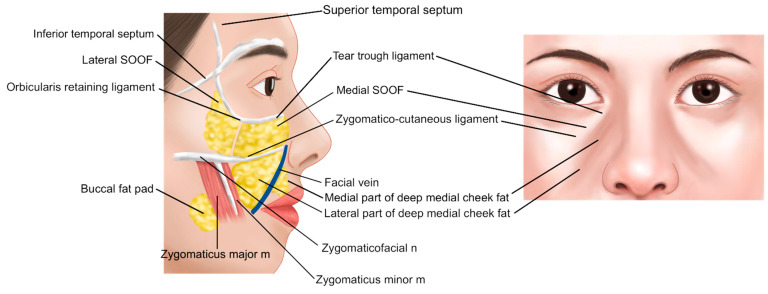
Infraorbital hollowness with loss of deep fat tissue.

As aging progresses, these symptoms tend to worsen. The skin loses its elasticity, leading to increased sagging and hollowing under the eyes, which exacerbates infraorbital hollowness. The primary factors contributing to this condition include the loss of volume in the deep fat layers beneath the arcus marginalis, particularly the sub-orbicularis oculi fat (SOOF) and the deep medial cheek fat, both of which are critical for supporting the central cheek (Figure 14) [21].

**Figure 15:**
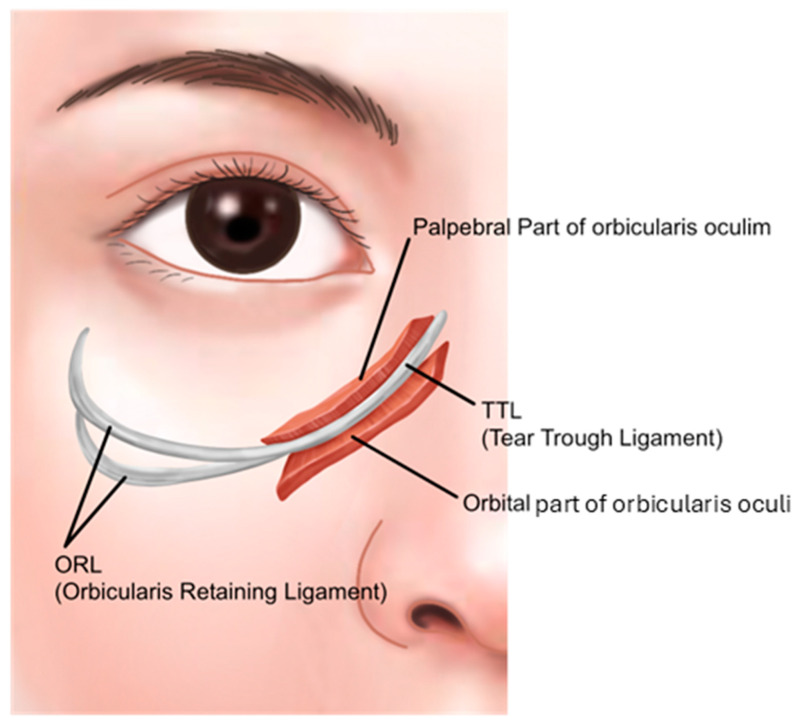
Tear trough and orbicularis retaining ligaments.

According to the anatomical studies by Dr. Wong [13], the TTL, located in the tear trough region, is a true osteocutaneous ligament extending from the maxilla bone to the skin. It is situated between the palpebral and orbital portions of the orbicularis oculi muscle. Medially, it originates just below the insertion of the medial canthal tendon at the anterior lacrimal crest and extends laterally to approximately the medial-pupil line, where it transitions into the orbicularis retaining ligament (ORL), which consists of upper and lower lamellae (Figure 15).

These ligamentous structures extend laterally from the area around the lateral canthus to the lateral orbital thickening. The inner portion of the orbicularis retaining ligament (ORL) connecting with the tear trough ligament (TTL) is relatively tight, while the outer portion is broader and more diffuse, leading to lateral hooding. Compared to the inner region, the outer portion is easier to release during undermining, providing sufficient space beneath the orbicularis oculi muscle for filler injection, making it a favorable area for such procedures.

Histological studies have indicated that both the ORL and TTL share similar ligamentous characteristics with the zygomatico-cutaneous ligament [22]. According to this study, the pronounced appearance of the groove on the medial side is due to the firm attachment of the muscle to the bone along the medial limbus line. As the muscle extends laterally from the midpupillary line, it loses its attachment to the bone, resulting in a less defined and more relaxed groove. The study further reported that histological examination did not reveal the presence of the tear trough ligament, which was thought to be situated between the orbital and palpebral parts of the orbicularis oculi muscle on the medial side. Similarly, the orbicularis retaining ligament, believed to exist on the lateral side, traversing the origin of the orbicularis muscle and connecting it to the orbital bone, was also not observed. Instead, the study found that there were no true ligaments within the muscle bundles of the orbicularis oculi, but rather a significant presence of fibrotic connective tissue structures, challenging previous theories about the anatomical structures in this area.

**Figure 16:**
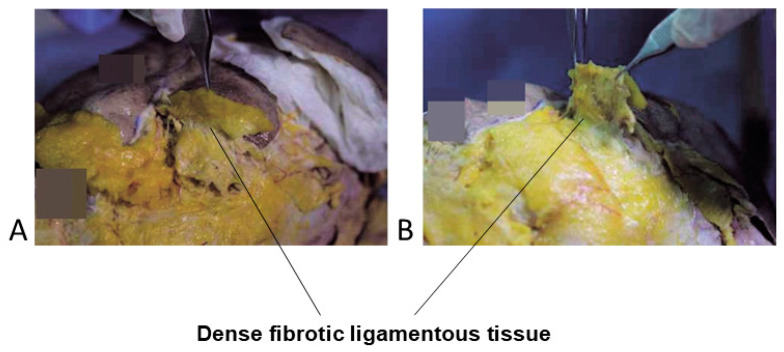
Tear trough ligament in anatomical study seen from the inferior (**A**) and lateral view (**B**).

The author, through multiple cadaver studies, has attempted to carefully dissect the sheet-like ligament in the tear trough area. However, in fixed cadavers, only a thin, paper-like ligamentous structure could be cleanly separated, whereas in fresh cadavers, dense fibrotic ligamentous tissue extending from the bone to the overlying fat layers was primarily observed, rather than a distinct ligament (Figure 16).

The author suggests that further cadaver studies and research are necessary to clarify whether this is a true ligament or a fibrotic connective tissue attachment. Regardless, the clinical approach to addressing the tear trough should consider the existence of a ligamentous structure connecting the orbital rim, orbital bone, orbicularis oculi muscle, and the overlying skin.

**Figure 17:**
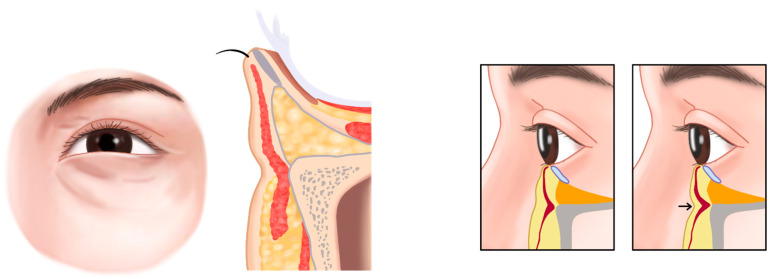
Tethering effect of the tear trough ligament causing depression.

When squinting or frowning, the tethering effect of the ligament connecting the orbicularis muscle to the skin causes the tear trough to become more pronounced. During filler injections to correct this area, it is advisable to inject below the ligament and push upwards, creating a lifting effect. However, in cases where the skin is significantly tethered, injecting only beneath the groove without releasing the ligament can cause the filler to bulge laterally, leaving the groove itself uncorrected and potentially exacerbating the deformity due to the enhanced tethering effect (Figure 17).

As individuals age, the tear trough and associated infraorbital hollowing become more pronounced due to the loss of skin elasticity, midface sagging, orbital fat protrusion, and the reduction in soft tissue volume around the eyes. This results in a more prominent hollowing effect as the orbicularis oculi muscle is pulled inward, while the tissues below the infraorbital hollow appear to sag due to the tethering effect of the TTL and ORL on the skin [1]. When these symptoms are present, a combination of filler injections and additional treatments may be necessary for effective correction.

**Figure 18:**
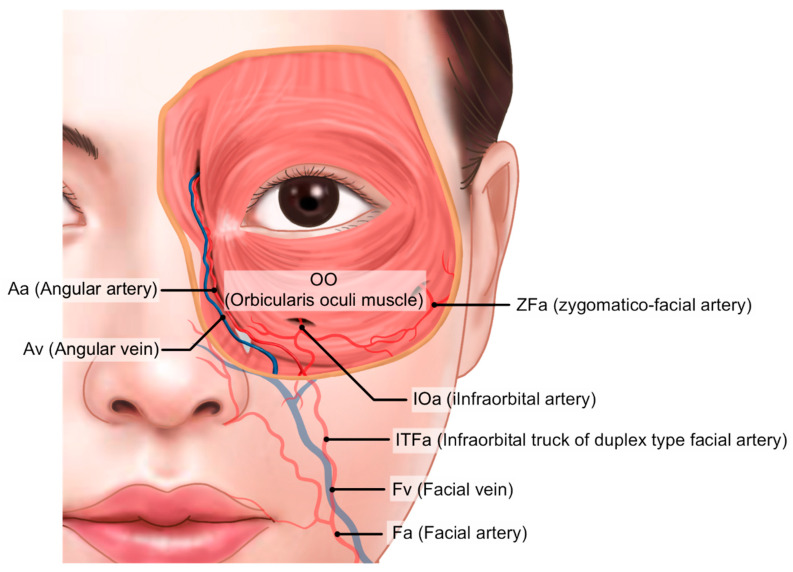
Main vessels of the infraorbital region.

For safe procedures, it is crucial to understand the important blood vessels running beneath the eyes. In more than 30% of Koreans, the nasojugal groove, which may be connected to the medial aspect of the midcheek groove, follows the path of the duplex-type facial artery’s infraorbital trunk—a branch of the facial artery that courses through the inferior orbit and lower eyelid region (Figure 18) [8,23]. Additionally, the facial vein, which travels deeper beneath the muscles, has a significantly large diameter and tends to dilate further when the patient is in a supine position. The infraorbital trunk of the duplex-type facial artery and the facial vein traverse the medial border of the deep fat layers (SOOF and deep medial cheek fat) and ascend along the medial border of the orbicularis oculi muscle’s orbital portion. Special care must be taken during procedures in this area to avoid vascular complications (Figure 18) [24].

In this region, the medial branch of the infraorbital artery, which originates from the maxillary artery (a branch of the external carotid artery), also traverses. This branch follows the path of the nasojugal groove and emerges superficially as it reaches the area where the nasojugal groove intersects with the tear trough (Figure 18) [25].

### 3.2. Procedure Method

**Figure 19:**
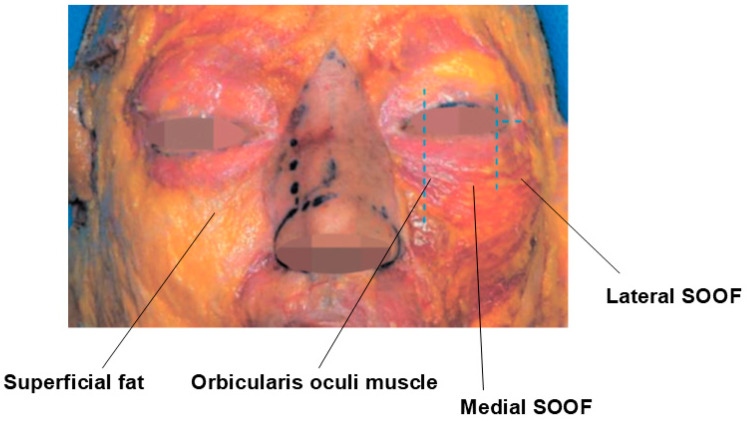
Medial and lateral SOOF.

From the anterior lacrimal crest near the inner canthus to the medial orbit, the orbicularis oculi muscle lies flat against the bone, with minimal or no intervening fat tissue, creating a ligamentous attachment. Beyond this region, within the tear trough, both superficial and deep malar fat are present above and below the orbicularis oculi muscle. The deep fat layer beneath the orbicularis oculi muscle is known as the SOOF, which is subdivided into medial SOOF and lateral SOOF. The medial SOOF spans from the medial limbus line to a vertical line through the lateral canthus, while the lateral SOOF extends from this vertical line to the boundary of the temporal fat pad, typically marked by a horizontal line from the lateral canthus (Figure 19).

The medial portion of the tear trough is characterized by tight muscular bands, with no SOOF layer beneath the muscle, making deep fat layer injections fundamentally impossible. Although some practitioners may attempt injections near the orbital bone, the muscle adheres tightly to the bone, leaving little space between them. Therefore, in practice, the filler is likely to be injected into or above the muscle itself.

For the medial portion of the tear trough, it is advisable to inject filler just above the periosteum within the muscle or along its boundary. For the lateral portion, where the SOOF layer exists, a soft filler (L’orient, Joonghun Pharmaceutical, Seoul, Republic of Korea) should be gently injected into the deep fat layer beneath the muscle along the tear trough’s boundary, lifting the depression from below. However, care must be taken not to inject excessive amounts into the deep layer of the lateral portion, especially in patients with dark circles, as this can exacerbate discoloration.

**Figure 20:**
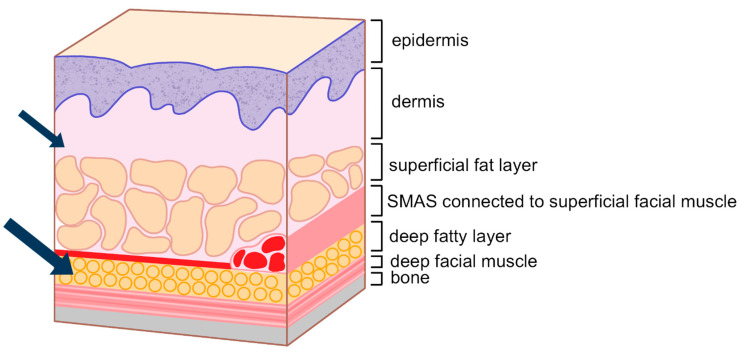
Injection planes: (1) submuscular injection of Juvederm Volift or Volbella the periorbital region (including SOOF and DMCF) for infraorbital groove and hollowness; (2) tear trough deformity treated within or above the muscular origin of orbicularis oculi muscle due to limited submuscular space in the medial portion; (3) subdermal injection of Juvederm Volbella or Skinvive for fine lines, addressing thin skin and smoothing irregular surfaces.

After restoring the deep layer beneath the orbicularis oculi muscle in the tear trough, any residual unevenness or hollowness can be corrected using a dual-plane technique. This involves injecting a very soft filler (L’orient, Joonghun Pharmaceutical, Seoul, Republic of Korea) superficially beneath the dermis above the orbicularis oculi muscle to smooth the surface (Figure 20).

When performing injections under the eyes, it is essential to be mindful of the numerous veins, including the inferior palpebral vein, which are prominent in this area. When using a needle, care must be taken to avoid these veins during the procedure. In cases where the hollowing is not severe or is limited to the medial portion of the tear trough, a needle can be effectively used. However, for overall volume restoration, it is safer to use a cannula to minimize the risk of vascular injury.

Key vascular structures in the lower eyelid region include the infraorbital artery, which emerges from the infraorbital foramen located 6–10 mm below the orbital margin along the medial pupillary line. Another significant artery, the zygomaticofacial artery, branches from the maxillary artery and exits from the zygomaticofacial foramen, typically situated 15–20 mm below the horizontal line extending from the lateral canthus. This foramen is located just below the lateral orbital rim margin (Figure 18).

**Figure 21:**
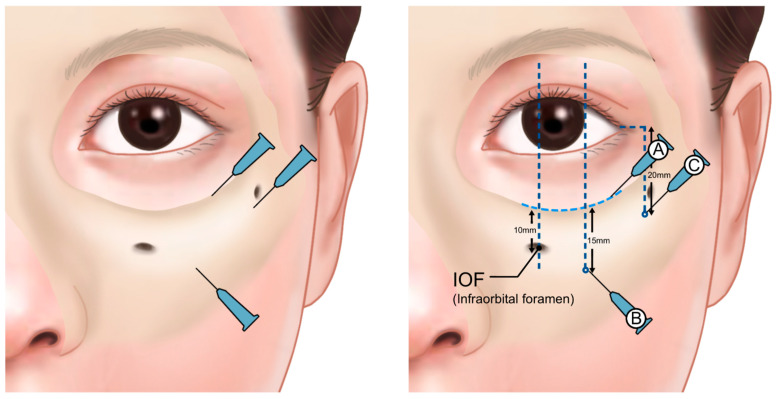
Injection entry points and techniques for needle and cannula. A—Injection around the orbital rim using linear threading and droplet techniques; B—cannula entry point for treating grooves; C—cannula entry point for treating hollowness. Directions toward the palpebromalar groove and nasojugal groove. Techniques include retrograde fanning, linear threading, and very slow release.

When using a cannula, it is recommended to avoid these vascular areas by creating a puncture site along the vertical line extending from the lateral limbus, approximately 15–20 mm below the orbital margin. If the procedure also involves correcting the midcheek hollow, a puncture site can be established 20 mm below the orbital margin along the vertical extension of the lateral orbital rim. A longer cannula can then be used to simultaneously address the under-eye area and the anterior cheek (Figure 21). This approach helps ensure a safe and effective procedure, minimizing the risk of complications such as vascular injury.

After establishing the puncture site, gently advance the cannula beneath the hollowed areas, using a fanning technique to evenly distribute the filler and the retrograde linear threading technique to smooth out the recessed grooves. Even if sufficient volume is restored, the medial portion of the tear trough near the inner canthus may still appear as a fine line due to the strong pull of the tear trough ligament on the skin. In such cases, additional injections of soft filler (L’orient, Joonghun Pharmaceutical, Seoul, Republic of Korea) into the upper subdermal layer, as previously described, can help reinforce the dermal tissue and smooth out the fine line.

**Figure 22:**
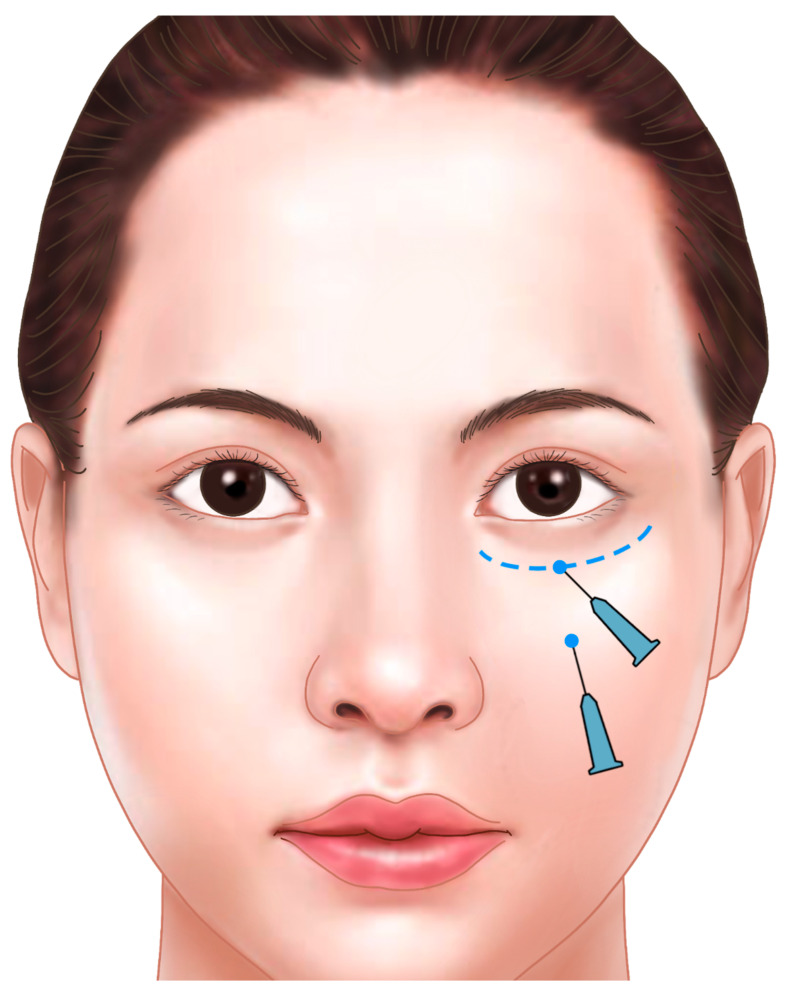
Injection techniques for superficial injections with a cannula or needle. Entry points and planes around the orbital rim for treating tear trough deformity and surface irregularities. Subdermal injection to augment fine depressions and smooth surfaces. Techniques include droplet technique and tenting technique using Juvederm Volbella or Skinvive (0.2–0.3 mL).

If the skin appears tightly adhered to the orbicularis oculi muscle, making the groove more pronounced when squinting, consider releasing the attachment site with the cannula before injecting the filler to achieve a smoother appearance. Performing a “pushing test”—where the skin is gently pushed upward—can indicate the need for release if the depression becomes more pronounced. For superficial corrections on the lateral side, use a needle or cannula to inject soft HA filler (L’orient, Joonghun Pharmaceutical, Seoul, Republic of Korea) into the subdermal layer beneath the dermis to finalize the procedure (Figure 22).

**Figure 23:**
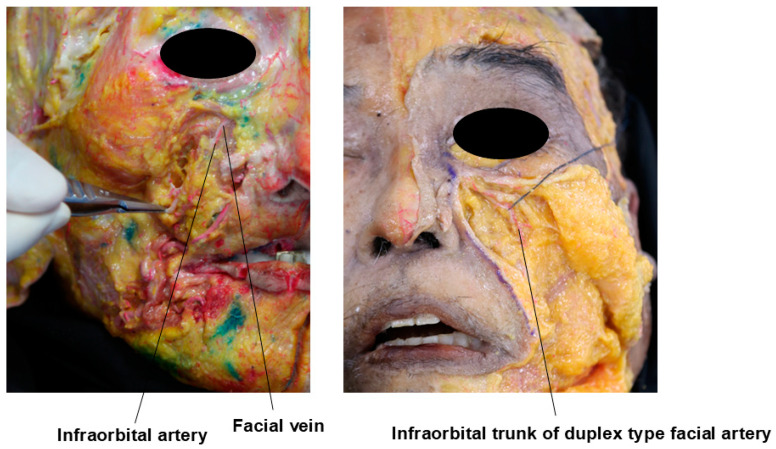
Main vessels on the nasojugal groove.

When treating the nasojugal groove, which forms between the medial part of the deep medial cheek fat and the lateral part of the SOOF, proceed with caution regarding the vascular structures that run along the groove. Follow the treatment guidelines for the tear trough when selecting injection points and performing the procedure (Figure 23).

For optimal results, the injection points for treating tear troughs and infraorbital hollowness should be planned while the patient is seated with their eyes open. As mentioned, when using a cannula, carefully design the entry point for smooth access. If using a needle, avoid visible veins on the skin’s surface to minimize the risk of vascular complications.

During injection, ensure precision by slowly injecting the filler beneath the skin while the patient keeps their eyes open, taking care to avoid forming any boluses. After injection, gently massage the area to ensure even distribution of the filler, preventing any irregular clumping. Be cautious to avoid displacing the filler into the orbital fat pad.

Given the potential for significant post-procedure swelling in the under-eye area, it may be advisable to apply a cooling pack for up to 24 h post-procedure to reduce excessive bruising or swelling. It is often better not to aim for perfect correction in the initial session but rather to achieve moderate improvement, with a follow-up session after approximately two weeks to finalize the treatment.

When performing filler injections in the tear trough or infraorbital hollowness, it is important to be mindful of potential complications such as bruising, swelling, tenderness, and hematoma. If high-viscosity fillers that absorb a lot of moisture are injected unevenly or in excessive quantities, they may clump and become visible through the skin. In particular, injecting too much filler superficially can result in the filler gel being visible through the skin (Tyndall effect) or creating uneven, bead-like protrusions. Therefore, it is crucial to avoid over-correction. Typically, it is recommended to limit the volume of filler injected to 0.3–0.5 mL per side in a single session (Table 2).

When a patient presents with both a midcheek groove and tear trough or infraorbital hollowness, it is important to inform them that treating the tear trough or infraorbital hollowness alone might make the midcheek groove appear more prominent, creating a band-like effect. Ideally, both conditions should be addressed simultaneously to achieve a balanced and harmonious outcome.

Patients with significant wrinkles or skin laxity in the lower eyelid area, extremely thin skin, severe hyperpigmentation, prominent bulging of the infraorbital fat pads, a history of severe allergies or edema around the eyes, or those who have undergone previous lower eyelid surgery or have scar tissue for other reasons may not respond well to filler treatment in the tear trough or infraorbital hollowness. These patients are at higher risk for complications, and the desired effects may not be fully achieved.

In such cases, it is crucial to consider whether additional treatments should be planned in conjunction with the filler procedure to optimize results and minimize risks (Table 3).

## 4. Discussion

The article addresses how to understand and correct lower eyelid aging through filler injections, focusing on anatomical features and age-related changes specific to this region.

The infraorbital groove and hollowness are complex anatomical conditions that significantly affect the esthetics of the periorbital region, particularly as individuals age. The primary cause of these changes is the progressive loss of volume in the deep fat pads, such as the sub-orbicularis oculi fat (SOOF) and malar fat pad, coupled with skin thinning and decreased elasticity [26,27,28]. These factors contribute to the deepening of the tear trough and the formation of infraorbital hollowness. Additionally, the descent of superficial fat pads and the presence of ligamentous structures like the tear trough ligament and orbicularis retaining ligament (ORL) exaggerate the groove and hollow appearance [7,18,22,29]. A precise understanding of these anatomical structures is essential for successful treatment, as they dictate the approach for filler placement and other corrective procedures.

Treatment strategies for infraorbital groove and hollowness require a careful balance between restoring volume and maintaining natural contours. Filler injections, especially hyaluronic acid-based fillers, are commonly used to address these issues by replenishing lost volume and softening the appearance of grooves. The injection technique must consider the different layers of tissue to avoid complications such as overfilling, bruising, or vascular injury. For example, in the tear trough region, the presence of the tear trough ligament requires a submuscular or supraperiosteal injection to lift the hollow effectively [7,12,16,17,19,30,31,32,33,34]. In contrast, for more superficial corrections, a subdermal filler placement can be used to smooth the surface and reduce the visibility of multiple eyelid lines. Understanding the role of the orbital rim, the transition between thin eyelid skin and thicker cheek skin, and the vascular structures is crucial to achieving optimal outcomes.

The success of filler treatments in the infraorbital region also depends on the patient’s unique anatomy and the severity of the hollowness. In some cases, volume restoration in the tear trough alone may lead to an unbalanced appearance, where untreated midcheek grooves or malar bags become more pronounced [35,36,37,38,39,40,41,42,43,44,45]. Therefore, a comprehensive approach addressing both the infraorbital and midcheek regions is often necessary to achieve esthetic harmony. Furthermore, while volume loss in the deep fat compartments plays a key role in periorbital aging, sagging of the superficial fat compartments also contributes significantly to the formation of infraorbital hollowness and contour irregularities. Since filler injections primarily address volume depletion rather than skin laxity or ptosis of the superficial fat, their effectiveness may be limited in cases with advanced soft tissue descent.

As such, filler treatment alone may not always achieve the desired rejuvenation, particularly in patients with significant skin laxity, severe superficial fat ptosis, or pronounced malar festoons. In these cases, adjunctive treatments such as thread lifting, laser resurfacing, radiofrequency-based tightening, or surgical interventions (e.g., lower blepharoplasty) may be required to achieve optimal results. Additionally, poly-D,L-lactic acid can serve as a biostimulatory adjunct, improving skin quality and firmness over time through collagen stimulation. Therefore, proper patient selection is crucial, as individuals with extensive periorbital aging changes may require a multimodal approach rather than filler treatment alone. A comprehensive assessment, clear patient communication, and a tailored treatment plan remain fundamental to achieving natural, long-lasting results.

## 5. Conclusions

In conclusion, this study highlights the critical role of anatomical knowledge in achieving safe and esthetically pleasing outcomes in the treatment of the infraorbital groove and hollowness. By utilizing filler injections tailored to the unique structural characteristics of the infraorbital region, including fat pad distribution, ligament attachments, and vascular considerations, practitioners can more effectively address age-related volume loss and achieve natural results. The comprehensive review of filler techniques and anatomical variations provides valuable insights for clinicians, emphasizing the importance of individualized treatment approaches. With the increasing demand for non-surgical periorbital rejuvenation, this study contributes essential guidelines to enhance procedural accuracy and patient safety.

## Figures and Tables

**Table 1:** Causes of infraorbital groove and hollowness.

1. Tear trough and orbicularis retaining ligaments
2. Difference in skin thickness and texture at lid-cheek junction
3. Difference in subcutaneous fat layer thickness at lid-cheek junction
4. Intraorbital fat bulging or depression
5. Decrease in deep fat (SOOF) inferior to arcus marginalis
6. Medial muscular band of OOM
7. Contraction of origin of LLSAN And LLS muscle

**Table 2:** Side effects of HA filler injection for the infraorbital groove and hollowness.

1. Bruising
2. Swelling
3.Tenderness
4. Hematoma
5. Visible irregularities (beading): especially when injecting superficially
6. Overcorrection
7. Accentuation of midcheek groove due to the change in the subcutaneous fat or the dermal components
8. Visible gel (Tyndall effect)

**Table 3:** Not recommended in superficial injection of the dermal fillers in the following situations.

1. Excessive wrinkling or excess skin
2. Very thin fine skin
3. Prominent pre-existing color change (dark circle)
4. Prominent orbital fat pads
5. History of severe allergy or swelling around the eye region
6. Previous eyelid surgery or scarring tissue

## Data Availability

Data are available by request to corresponding author.
